# La retractación de artículos científicos: un compromiso con la integridad, la transparencia y la confianza en la investigación en salud y biomedicina

**DOI:** 10.7705/biomedica.8710

**Published:** 2026-10-16

**Authors:** Luis Alberto Gómez G.

**Affiliations:** 1 Editor en jefe, revista Biomédica Instituto Nacional de Salud Bogotá, D.,C. Colombia Instituto Nacional de Salud Instituto Nacional de Salud Bogotá, D.,C. Colombia

La ciencia avanza mediante un proceso continuo de generación, contraste, revisión y corrección del conocimiento. Ningún sistema de producción científica está exento de errores y, por ello, la capacidad de reconocerlos y corregirlos constituye una condición esencial para preservar la credibilidad de la ciencia. En este contexto, la retractación de un artículo no debería interpretarse, por sí misma, como un fracaso del sistema editorial ni como un juicio sobre sus autores. Cuando está debidamente sustentada y comunicada, constituye uno de los mecanismos mediante los cuales la comunidad científica protege la integridad y la confiabilidad del registro publicado ^1^^-^^3^.

Esta responsabilidad adquiere especial importancia en las ciencias de la salud y en la biomedicina. Los resultados publicados pueden orientar decisiones clínicas, recomendaciones diagnósticas o terapéuticas, revisiones sistemáticas y metaanálisis, políticas públicas, asignación de recursos y nuevas líneas de investigación. Por ello, el mantener en la literatura resultados cuya validez ha sido cuestionada de manera sustancial puede tener consecuencias que trascienden el ámbito académico. El corregir oportunamente el registro científico representa una responsabilidad frente a los investigadores y profesionales de la salud, pero también frente a los pacientes, las comunidades y a quienes toman decisiones basadas en la evidencia.

Las revistas científicas son custodias del registro académico y su responsabilidad no termina con la publicación de un manuscrito. También deben contar con procedimientos claros para actuar cuando, después de la publicación, surge información que afecta la exactitud, la validez o la integridad del contenido. Esto requiere distinguir entre una corrección, una expresión de preocupación, una retractación, una eventual retractación con republicación y, en circunstancias excepcionales, la remoción de un contenido ^1^^-^^4^.

El *Committee on Publication Ethics* (COPE) establece que el propósito fundamental de la retractación es corregir la literatura y preservar su integridad, no castigar a los autores. Una publicación puede ser considerada para retractación cuando existe evidencia clara de que sus resultados no son confiables debido, entre otras razones, a errores que afectan sustancialmente sus resultados o conclusiones, fabricación o falsificación de datos, plagio, publicación redundante, utilización de materiales o datos sin la debida autorización, investigación que no cumple los requerimientos éticos, alteraciones del proceso de revisión por pares o situaciones legales o de conflicto de interés que comprometen de manera significativa la interpretación del trabajo ^1^.

Este criterio es importante porque la confiabilidad de la publicación, y no únicamente la atribución de responsabilidades, debe orientar la decisión editorial. Una retractación puede ser necesaria tanto cuando se identifican problemas relacionados con la integridad de la investigación como cuando se reconocen errores involuntarios que comprometen de manera sustancial la validez de los resultados o de las conclusiones. Los problemas que afectan la confiabilidad de una publicación pueden derivarse de errores honestos, limitaciones metodológicas o analíticas, así como de conductas que comprometen la integridad de la investigación; aunque estas situaciones tienen naturaleza diferente, todas deben evaluarse en función de su impacto sobre la validez de los resultados y conclusiones. Por ello, la retractación y la responsabilidad individual no son conceptos equivalentes. El reconocer esta diferencia contribuye a evitar que se estigmatice y favorece una cultura en la que los investigadores pueden comunicar de manera transparente los errores identificados después de la publicación.

Cuando los propios autores detectan una equivocación relevante y participan activamente en su corrección, esta actuación debe entenderse como parte de una cultura de integridad científica. COPE recomienda que las comunicaciones editoriales describan con precisión las circunstancias que motivaron la modificación del registro y permitan comprender, cuando sea posible, la naturaleza del problema identificado ^1^^,^^2^.

No todo error justifica una retractación. Cuando el problema afecta una parte delimitada del artículo y los principales resultados y conclusiones mantienen su validez, una corrección puede ser suficiente. El *International Committee of Medical Journal Editors* (ICMJE) recomienda que las correcciones se publiquen oportunamente, describan de forma clara las modificaciones realizadas, permanezcan vinculadas con el artículo original y sean comunicadas a los sistemas de indexación. Asimismo, debe preservarse la historia de las modificaciones para garantizar la trazabilidad del registro científico ^3^.

El ICMJE contempla también la retractación con republicación, o reemplazo, en determinadas circunstancias. Esta alternativa puede considerarse cuando un error involuntario, por ejemplo, una clasificación errónea o un cálculo incorrecto, modifica de manera sustancial los resultados, su interpretación o las conclusiones, aunque la investigación subyacente mantiene su validez científica. Tras una nueva evaluación editorial y científica, la versión corregida puede publicarse nuevamente acompañada de una explicación transparente de lo ocurrido y de las modificaciones hechas ^3^. Esta posibilidad refuerza una concepción de la corrección científica orientada a preservar la precisión, la transparencia y la confiabilidad del registro publicado.

También existen situaciones en las que surgen dudas relevantes sobre una publicación, pero la información disponible todavía no permite adoptar una decisión definitiva. En estos casos, una expresión de preocupación puede ser apropiada para informar a los lectores mientras se completa la evaluación institucional o editorial, sin anticipar sus resultados. Una vez obtenida suficiente información, esta comunicación puede conducir a una corrección, a una retractación o a una aclaración de que las dudas han sido resueltas ^1^^-^^3^.

Las decisiones posteriores a la publicación deben adoptarse mediante procedimientos coherentes, imparciales y documentados. Esto implica evaluar la información disponible, solicitar explicaciones a los autores cuando corresponda y, cuando la naturaleza del caso lo requiera, establecer comunicación con las instituciones responsables de adelantar las evaluaciones pertinentes. Las instituciones y las revistas cumplen funciones diferentes, pero complementarias: las primeras examinan los aspectos relacionados con el desarrollo de la investigación, mientras que las revistas deben determinar las medidas necesarias para preservar la integridad de aquello que han publicado ^1^^,^^2^.

El debido proceso editorial supone brindar a las partes involucradas una oportunidad razonable para responder a los cuestionamientos y garantizar una valoración objetiva, documentada e imparcial de la información disponible. No obstante, la falta de consenso entre todos los autores no debería impedir una retractación cuando los editores cuentan con elementos lo suficientemente sólidos para concluir que los resultados, las conclusiones o la integridad del artículo están comprometidos de manera sustancial. De igual forma, si una evaluación se prolonga y persisten dudas relevantes sobre la validez del trabajo publicado, resulta importante mantener informados a los lectores; en estas circunstancias, una expresión de preocupación puede constituir una medida provisional apropiada ^1^^-^^3^.

La transparencia debe reflejarse también en la manera como se comunica una retractación. De acuerdo con COPE e ICMJE, el aviso debe identificarse claramente, señalar quién adopta la decisión y explicar de forma objetiva y comprensible las razones que la sustentan. La comunicación de una retractación debe ser proporcional a la naturaleza del problema identificado, distinguiendo claramente si este se relaciona con errores, limitaciones metodológicas, incumplimientos éticos o conductas que comprometan la integridad científica, y evitando inferencias sobre intencionalidad cuando estas no hayan sido establecidas mediante los procedimientos correspondientes. Asimismo, debe permanecer vinculado con el artículo original. Este, por regla general, debe continuar disponible para preservar la trazabilidad del registro científico, pero con una indicación visible e inequívoca de su condición de retractado. El aviso, a su vez, debe enlazarse con el artículo original y ser de libre acceso ^1^^,^^3^.

La retractación no implica, por regla general, la eliminación del artículo del registro científico. El mantener disponible la publicación retractada, debidamente identificada y vinculada con su aviso, permite conservar la transparencia y la historia del proceso editorial. La remoción completa de un contenido debería limitarse a situaciones excepcionales, como aquellas derivadas de obligaciones legales, vulneraciones graves de la privacidad o riesgos relevantes para la salud pública. Aun en estos casos, es recomendable conservar, cuando sea posible, la información bibliográfica esencial y comunicar claramente las razones de la remoción ^1^^,^^4^.

En el entorno digital actual, la corrección del registro científico no se limita al sitio web de la revista. Las publicaciones circulan a través de bases bibliográficas, repositorios, índices, gestores de referencias, plataformas de descubrimiento y otros sistemas de información. Por esta razón, el estado editorial de un artículo debe comunicarse de manera consistente en los distintos entornos en los que se encuentra disponible.

En este contexto, la *National Information Standards Organization* (NISO), mediante la *Recommended Practice NISO RP-45-2024, Communication of Retractions, Removals, and Expressions of Concern* (CREC), recomienda estandarizar la creación, transmisión y visualización de los metadatos asociados con las retractaciones, las remociones y las expresiones de preocupación. El propósito es garantizar que el estado editorial de una publicación pueda identificarse de manera clara, constante y trazable, tanto por los lectores como por las bases de datos, los índices bibliográficos, los repositorios y los sistemas automatizados que procesan literatura científica ^4^.

En consecuencia, una política actual de retractaciones debe contemplar tanto la decisión editorial como su adecuada comunicación y trazabilidad. El artículo retractado y su aviso deben permanecer vinculados; su condición editorial debe identificarse claramente en las diferentes versiones disponibles, y los metadatos transmitidos a bases de datos e índices bibliográficos deben reflejarla de forma inequívoca ^3^^,^^4^. Una comunicación incompleta puede favorecer el que una publicación retractada continúe siendo citada o que sus resultados se incorporen a nuevos estudios, revisiones o decisiones clínicas sin considerar adecuadamente su estado editorial.

El aumento del número de retractaciones registrado en la literatura científica debe interpretarse con cautela. Por sí solo, este incremento no permite concluir que existe un deterioro de las prácticas de investigación. También puede reflejar una mayor capacidad de las revistas, las instituciones y las comunidades científicas para identificar inconsistencias de los datos, alteraciones de imágenes, similitudes textuales indebidas, deficiencias en los procesos de revisión por pares y otras situaciones que anteriormente podían pasar inadvertidas. A ello se suman una evaluación pospublicación más activa y el desarrollo de herramientas especializadas para examinar la integridad de los contenidos. Las retractaciones deben comprenderse, por tanto, como parte de un sistema más amplio orientado a preservar la integridad, la transparencia y la calidad de la investigación y de sus resultados científicos, tecnológicos y de innovación.

El crecimiento sostenido de la producción científica plantea, al mismo tiempo, nuevos desafíos para la comunicación académica. La presión por publicar, los sistemas de incentivos que pueden privilegiar la cantidad sobre la calidad, algunas prácticas editoriales poco rigurosas, la producción organizada de manuscritos de baja confiabilidad, las interferencias en los procesos de revisión por pares y el uso inadecuado o poco transparente de herramientas basadas en inteligencia artificial pueden afectar diferentes etapas de la investigación y de su publicación. La respuesta editorial, por ello, no debería concentrarse únicamente en detectar problemas después de la publicación, sino también en fortalecer la prevención, la formación en integridad científica y las buenas prácticas editoriales.

La prevención requiere acciones a lo largo de todo el proceso de investigación y publicación. Entre ellas se encuentran la formación continua de autores, revisores y editores; una adecuada gestión y conservación de los datos; la transparencia metodológica; la declaración de posibles conflictos de interés; el uso apropiado de guías de reporte; la identificación clara de las contribuciones de autoría y el empleo responsable y transparente de tecnologías emergentes. La evaluación editorial inicial y final, una revisión por pares rigurosa y acorde con las características del manuscrito, las herramientas para identificar inconsistencias y una cuidadosa corrección de estilo pueden contribuir a reconocer oportunamente problemas antes de que el artículo se incorpore de manera definitiva al registro científico.

Aun con procedimientos preventivos sólidos, seguirá siendo necesario disponer de mecanismos para corregir la literatura después de su publicación. La ciencia es un proceso dinámico y acumulativo, y algunas limitaciones, errores o inconsistencias solo se hacen evidentes cuando los resultados son reproducidos, los datos se reanalizan, los hallazgos se comparan con nueva evidencia o las conclusiones se examinan a la luz de conocimientos posteriores. La evaluación posterior a la publicación debe entenderse, en este sentido, como una continuidad natural del proceso de revisión científica y como una herramienta para preservar la calidad y confiabilidad del registro científico.

La integridad de ese registro es una responsabilidad compartida. Autores, revisores, editores, instituciones académicas, comités de ética, financiadores, bases bibliográficas y lectores forman parte de un ecosistema interdependiente en el que cada actor cumple una función. Las instituciones tienen una responsabilidad particular en la evaluación de situaciones que puedan comprometer la integridad de la investigación; las revistas, en valorar sus implicaciones para la literatura publicada y adoptar las medidas editoriales correspondientes; y los sistemas de información, en garantizar que esas decisiones se comuniquen y reflejen de manera correcta, oportuna y trazable ^1^^-^^4^.

Para una revista científica, el contar con políticas claras de corrección y retractación va más allá del cumplimiento de estándares editoriales internacionales. Refleja la manera como asume su responsabilidad frente a la calidad, la integridad y la confiabilidad del conocimiento que publica. Su solidez no depende de que el registro permanezca inalterado, sino de su capacidad para reconocer las situaciones que requieren revisión, analizarlas con independencia y responder mediante medidas proporcionales, transparentes y debidamente documentadas.

La retractación debe entenderse, por tanto, como un mecanismo de corrección del registro científico. No corresponde aplicarla cuando una corrección permite resolver adecuadamente el problema identificado y preservar la validez de los principales resultados y conclusiones. La decisión debe centrarse en establecer si la publicación continúa ofreciendo información lo suficientemente confiable para formar parte del registro científico y, cuando ello no sea posible, adoptar la medida editorial que permita corregirlo de manera clara y transparente.

En una época caracterizada por la circulación rápida y global de la información y por la creciente influencia de la investigación biomédica en la práctica clínica, la salud pública y la toma de decisiones, el preservar la confianza en la literatura científica constituye una responsabilidad central. Esa confianza no se sustenta en considerar la ciencia como un proceso exento de errores, sino en disponer de mecanismos capaces de reconocer sus limitaciones, revisar críticamente la evidencia y corregir el registro cuando sea necesario.

La fortaleza de una revista científica radica, en última instancia, tanto en la calidad de los trabajos que publica como en su capacidad para responder con rigor, transparencia y oportunidad cuando un contenido requiere ser revisado o corregido. En las ciencias de la salud y en la biomedicina, este compromiso adquiere una relevancia particular: el preservar la integridad de la literatura contribuye a fortalecer la investigación, sustentar mejores decisiones clínicas y de salud pública y proteger, en último término, el bienestar de las personas y de las comunidades.
